# Acute Coronary Syndrome in an Anomalous Left Main Coronary Artery

**DOI:** 10.7759/cureus.61971

**Published:** 2024-06-08

**Authors:** Anjali Prakash, Sunny Goel

**Affiliations:** 1 Internal Medicine, Icahn School of Medicine at Mount Sinai South Nassau, New York, USA; 2 Cardiovascular Disease, Mount Sinai Hospital, New York, USA

**Keywords:** acute coronary syndrome, anomalous left coronary artery, right sinus of valsalva, anomalous coronaries, anomalous left main coronary artery, coronary vessel anomaly

## Abstract

Anomalous origin of the left main coronary artery (LMCA) from the right sinus of Valsalva (RSV) is a rare congenital anomaly that can cause myocardial ischemia, ventricular arrhythmia, and sudden cardiac death. We report the case of a 74-year-old male with a history of hyperlipidemia and hypertension who presented with non-ST elevation myocardial infarction (NSTEMI). On coronary angiogram, the patient was found to have LMCA originating from the RSV and a 90% stenosis of the left anterior descending (LAD) artery. The patient subsequently underwent computed tomography angiography (CTA) to assess the course of the LMCA, which was found to be intramyocardial with no compression. The echocardiogram reported a reduced ejection fraction (EF) of 40% and wall motion abnormalities in the anterior wall. The patient had a successful staged percutaneous coronary intervention (PCI). The patient on follow-up reported no symptoms and EF improved to 50%. Anomalous origin of the LMCA from the RSV is a rare but potentially life-threatening congenital anomaly. Once the course of the anomalous artery is established, immediate reperfusion using PCI is being increasingly used in place of surgical unroofing, noting a change in clinical practice. Further research is needed to determine the optimal treatment for this anomaly and to improve the long-term outcomes of affected individuals.

## Introduction

Congenital anomalies of coronary arteries are rare; the most critical of which is the left main coronary artery (LMCA) originating from the right sinus of Valsalva (RSV). This anomaly can cause myocardial ischemia, cardiac arrhythmias, and sudden cardiac death. Thus, early diagnosis and intervention are crucial for better patient prognosis. The mechanism of coronary ischemia in these patients is not well understood. The leading hypothesis among other theories suggests compression of the smaller and slit-like ostium of LMCA, leading to loss of blood flow during increased flow states [1].

Diagnosis of this anomaly can be made using non-invasive imaging techniques such as computed tomography angiography (CTA), magnetic resonance imaging (MRI), and echocardiography. Invasive coronary angiography can be used to confirm the diagnosis. Due to a lack of strong data, current guidelines recommend surgical interventions to treat these conditions (Class 1 recommendation) [2], but percutaneous coronary interventions are being increasingly noted to have reliable outcomes as well. Further research is needed to optimize the treatment for this anomaly and utilize less invasive approaches when feasible.

## Case presentation

**Figure 1:**
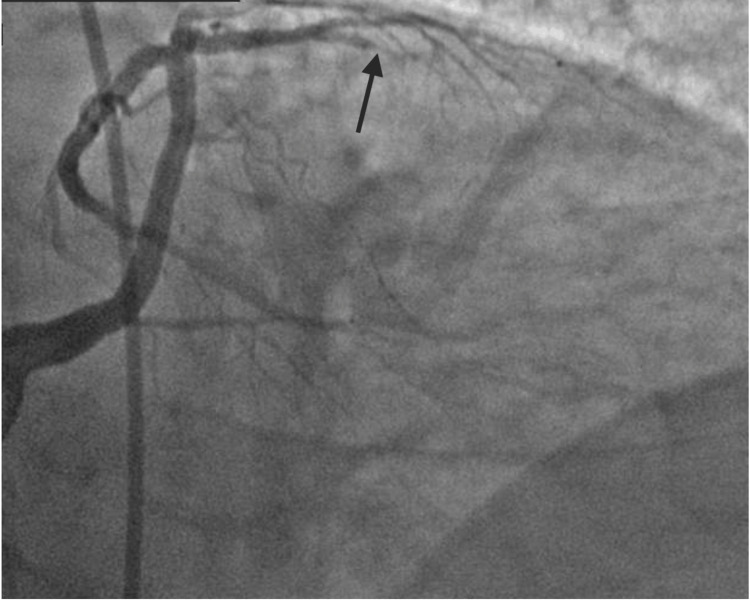
Coronary catheterization showing anomalous LMCA and significant stenosis of the mid-LAD artery (black arrow) LMCA: left main coronary artery; LAD: left anterior descending

A 74-year-old male with a history of hyperlipidemia and hypertension presented to the emergency room with weakness, chest tightness, and abdominal pain for two days. EKG on arrival showed normal sinus rhythm without any ST elevations. His troponin enzymes were elevated. The echocardiogram showed moderate to severe left ventricular systolic dysfunction with an ejection fraction (EF) between 30% and 35% and wall motion abnormalities in the anterior wall. Diagnostic catheterization revealed an anomalous large LMCA originating from the right cusp with significant stenosis in the mid-left anterior descending (LAD) artery (Figure 1, Video 1).

**Video 1:** Coronary catheterization showing an anomalous LMCA and significant stenosis of mid-LAD artery LMCA: left main coronary artery; LAD: left anterior descending

**Figure 2:**
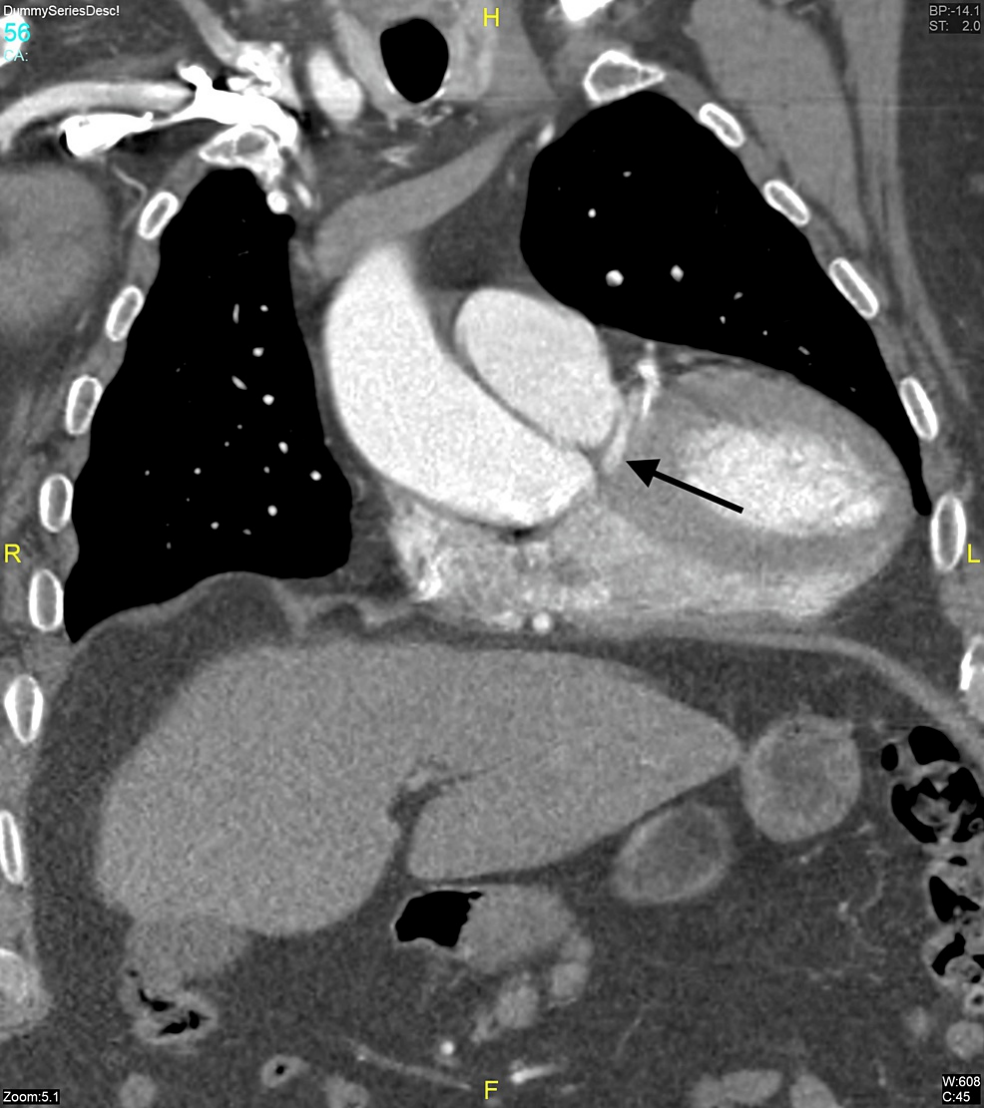
CT angiography (coronal view) showing the septal course of the LMCA (black arrow) LMCA: left main coronary artery

**Figure 3:**
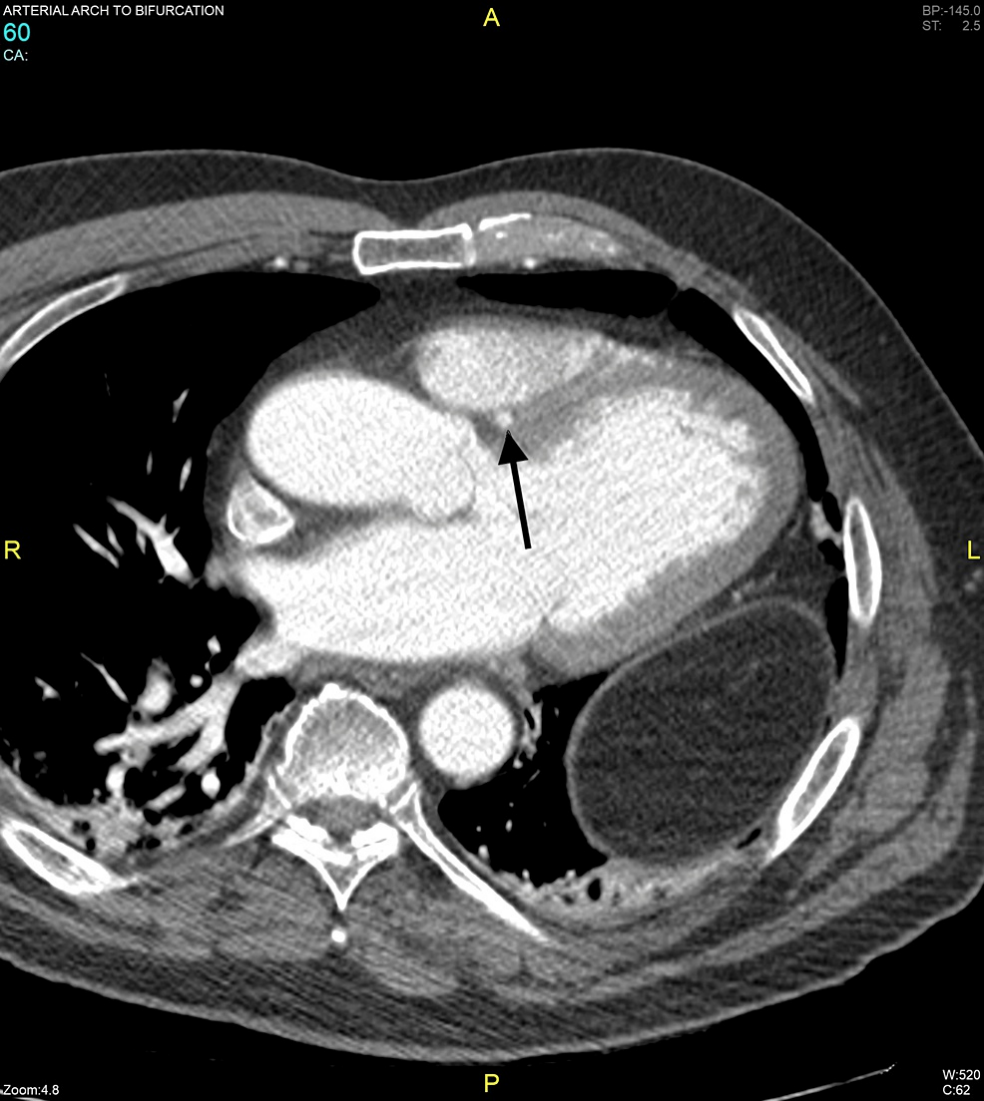
CT angiography (axial view) showing the septal course of the LMCA (black arrow) LMCA: left main coronary artery

The patient was sent for CT angiography to evaluate the course of LMCA. The LMCA had a septal course with no evidence of intra-arterial or intramyocardial/subendocardial compression (Figure 2, Figure 3).

**Figure 4:**
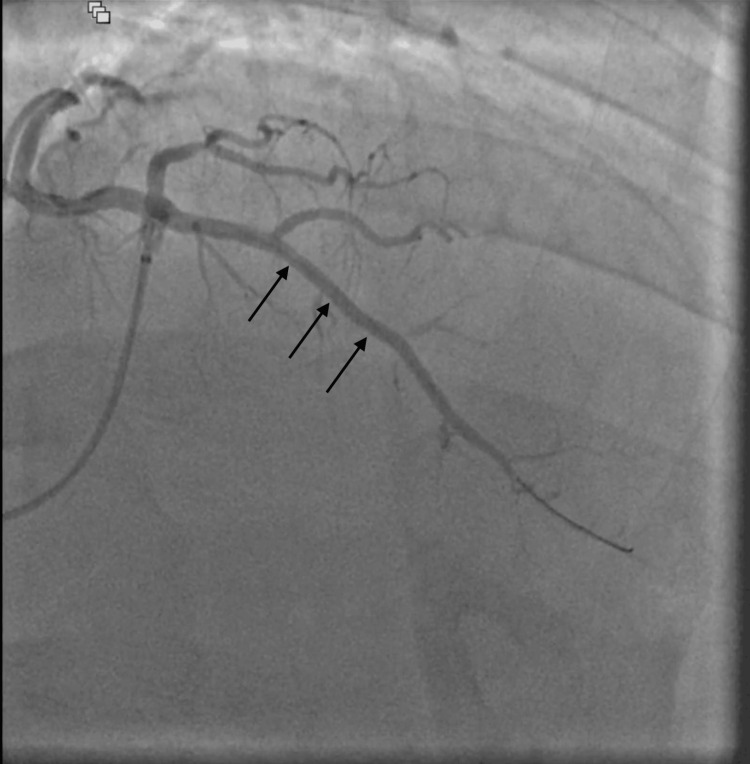
Coronary catheterization showing the return of perfusion after successful PCI (black arrows) of mid-LAD artery PCI: percutaneous coronary intervention; LAD: left anterior descending

The patient underwent staged percutaneous coronary intervention (PCI) with successful stent placement in the mid-LAD artery (Figure 4, Video 2). The patient was discharged on aspirin, clopidogrel, statin, and beta-blocker therapy. Follow-up echo after three months showed complete recovery of EF and was asymptomatic on follow-up.

**Video 2:** Coronary catheterization showing a return of perfusion after successful PCI of the mid-LAD artery PCI: percutaneous coronary intervention; LAD: left anterior descending

## Discussion

The prognosis of patients with an anomalous origin of the LMCA from RSV depends on various factors, including the severity of the anomaly, the presence of associated coronary artery anomalies, and the presence of symptoms. Early diagnosis and intervention can significantly improve the prognosis of affected individuals [3]. We recommend evaluation of anomalous coronary artery course using CT angiography to rule out possible compression, which might require further surgical management.

The optimal definitive treatment of this congenital anomaly is not fully understood. Due to a lack of data and the inability to predict sudden cardiac death, the latest guidelines recommend surgical intervention (class 1 recommendation) for all patients with LMCA from RSV with ischemia [2], especially if LMCA has an intramural course [1]. Surgery, including coronary artery bypass grafting (CABG) and re-implantation of the LMCA, is the most commonly performed intervention. However, percutaneous coronary intervention (PCI) has also been reported as a feasible treatment option. Similar to our case, a retrospective study of 35 patients with an anomalous right coronary artery (RCA) arising from the left sinus of Valsalva (LSV) who underwent PCI between 2009 and 2023, found that PCI was technically successful in the 35 patients and observed favorable long-term clinical follow-up in 80% of the cases [4]. Further research is needed to determine the optimal treatment for this anomaly and to improve the long-term outcomes of affected individuals.

## Conclusions

Anomalous origin of the LMCA from RSV is a rare but potentially life-threatening congenital anomaly that requires early diagnosis and appropriate management. In patients presenting with acute coronary syndrome and found to have an anomalous origin of the LMCA, PCI is a feasible treatment option once the course of the anomalous artery is visualized. Further research is needed to establish guidelines, to determine the optimal treatment for this congenital disease, and to improve patient outcomes.
